# Dermoscopy Features of Acquired Perforating Dermatosis Among 39 Patients

**DOI:** 10.3389/fmed.2021.631642

**Published:** 2021-04-08

**Authors:** Wenju Wang, Yansen Liao, Lixin Fu, Bei Kan, Xiaodong Peng, Yonghong Lu

**Affiliations:** ^1^Department of Dermatovenereology, Chengdu Second People's Hospital, Chengdu, China; ^2^Department of Oncology, Chengdu Second People's Hospital, Chengdu, China

**Keywords:** dermoscopy, acquired perforating dermatosis, characteristic, new dermoscopic features, clinical diagnosis

## Abstract

In this report, we concluded there are four dermoscopic features of APD including a yellow-brown homogeneous structureless area in the center of the lesion, dotted and linear vessels distribution radially and a dam shape uplift at the periphery, as well as a white irregular ring surrounding the lesion. There are three features, including the yellow-brown homogeneous structureless area in the center of the lesion, the dotted and linear vessels distribution radially and the white irregular ring surrounding the lesion were correspond to the report of Emma Ormerod et al.These features are also similar to those previously discribed in three separated reports of seven cases with APD. In our report, we found a new dermoscopic features: the dam shape uplift at the periphery. These finding may be contributed to improve the rate of clinical diagnosis of APD.

Acquired perforating dermatosis (APD) is a penetrating skin disease characterized by the excretion of denatured collagen from the epidermis. The most common clinical symptom of APD is pruritus. The lesions are distributed widely and characterized by umbilicated papules and plaques with central crusted ulceration (1). The histopathological presentation of APD often shows local epidermal erosion and loss, homogenized denaturation of dermal superficial fibers, and denaturated fiber bundles penetrating the epidermis (2, 3). MASSON's trichrome and elastic fiber stain showed collagen fibers penetrating the epidermis in the dermis. APD is a rare disorder and the clinical presentation can vary from patient to patient, and it is difficult to differentiate clinically from prurigo nodularis, solar dermatitis, or other diseases. The real diagnosis is often missed or delayed.

Dermoscopy is a non-invasive technique that is used to aid the diagnosis of skin tumors and other inflammatory dermatoses (4, 5) In our clinical work, we found the APD is characteristic under dermoscopy. To the best of our knowledge, the dermoscopic features of APD have been described in three previous case reports (6–8). Here we analyze and report the dermoscopic feature in a series of 39 patients with APD.

We identified 39 patients (19 male and 20 female) with a diagnosis of APD in our department over the past 4 years. The age of these patients ranged from 18 to 65 years, the average age was 48.5 years. All of the patients were diagnosed by clinical (Figure 1) and histopathological examination (Figure 2).

**Figure 1:**
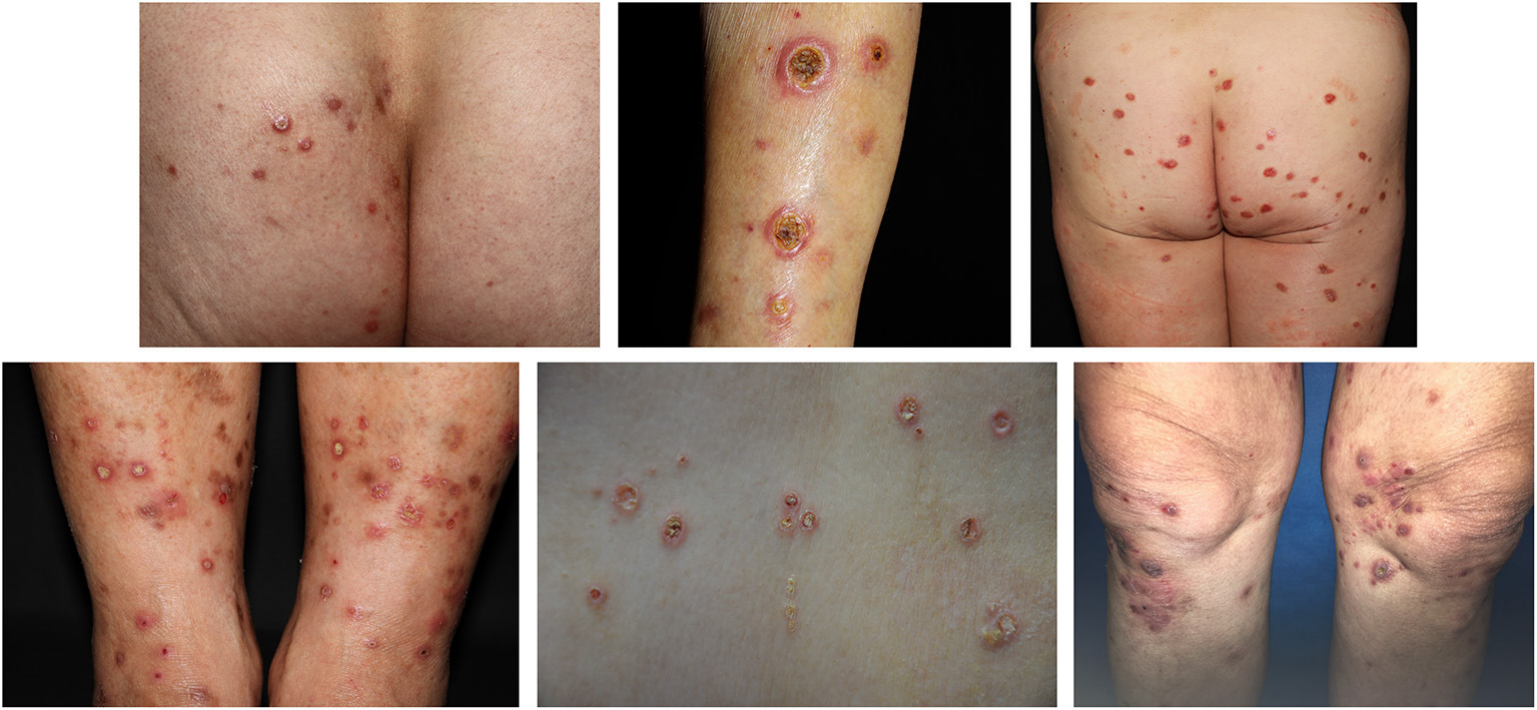
The clinical presentation of an APD patient. Skin lesions were widely distributed all over the trunk and limbs, the main manifestations were keratinized purplish-red papules or ulcers, about rice to bean size. The center of most papules was navel concave, the surface was a yellow adhesive exfoliated scab shell surrounded by a purplish-red halo. The edge of the ulcer was levee shaped and palpated with moderate hardness.

**Figure 2:**
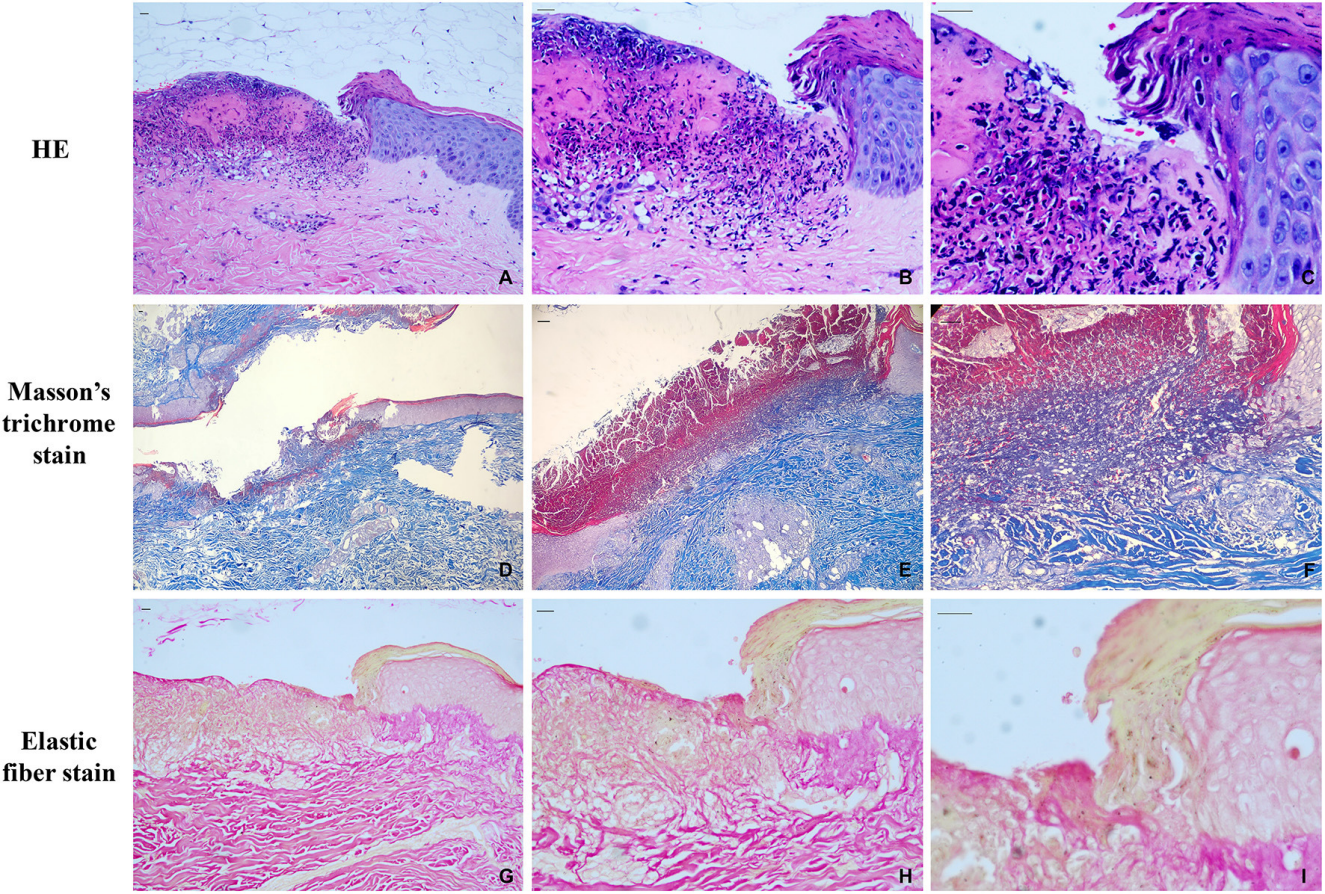
Histopathological features at initial presentation. **(A)** the HE staining of APD patients (10x); **(B)** the HE staining of APD patients (200x); **(C)** the HE staining of APD patients (400x); **(D)** Masson trichrome staining (40x); **(E)** Masson trichrome staining (100x); **(F)** Masson trichrome staining (200x); **(G)** elastic fiber staining (100x); **(H)** elastic fiber staining (200x); **(I)** elastic fiber staining (400x). Scale bars, 200 μm.

We analyzed the dermoscopy pictures of all 39 patients (Table 1). Dermoscopic images revealed that there are nine features: (1) a yellow-brown homogeneous structureless area in the center of the lesion were found in 38 of the 39 cases, the accrue rate was about 97.43% (Figure 3A); (2) dotted and linear vessel distribution radially were found in 38 of the 39 cases, the accrue rate was about 97.43% (Figure 3B); (3) a dam shape uplift at the periphery of the lesion was found in 37 of the 39 cases, the accrue rate was about 94.87% (Figure 3C); (4) a white irregular halo surrounding the lesion was found in 34 of the 39 cases, the accrue rate was about 87.18% (Figure 3D); (5) a thin scale ring surrounding the lesion was found in 21 of the 39 cases, the accrue rate was about 53.84% (Figure 3E); (6) a pink background in the lesion were found in 16 of the 39 cases, the accrue rate was about 41.02% (Figure 3F); (7) we found an an orange background in the lesion in 12 of the 39 cases, the accrue rate was about 30.77% (Figure 3G); (8) we found a red background in the lesion in 11 of the 39 cases, the accrue rate is about 28.20% (Figure 3H); (9) a pigment structure surrounding the lesion were found in 3 of the 39 cases, the accrue rate is about 7.69% (Figure 3I).

**Table 1:** Dermoscopic features observed in 39 ARPC patients with relative frequencies.

**Dermoscopic features**	***N* (Total 39)**	**Rate (%)**
yellow-brown homogeneous structureless area in the center	38	97.43
dotted and linear vessels distribution radially	38	97.43
adam shape uplift at the periphery	37	94.87
white irregular halo surrounding the lesion	34	87.18
thin scale ring surrounding the lesion	21	53.84
pink background	16	41.02
orange background	12	30.77
red background	11	28.20
pigment structure surrounding the lesion	3	7.69

**Figure 3:**
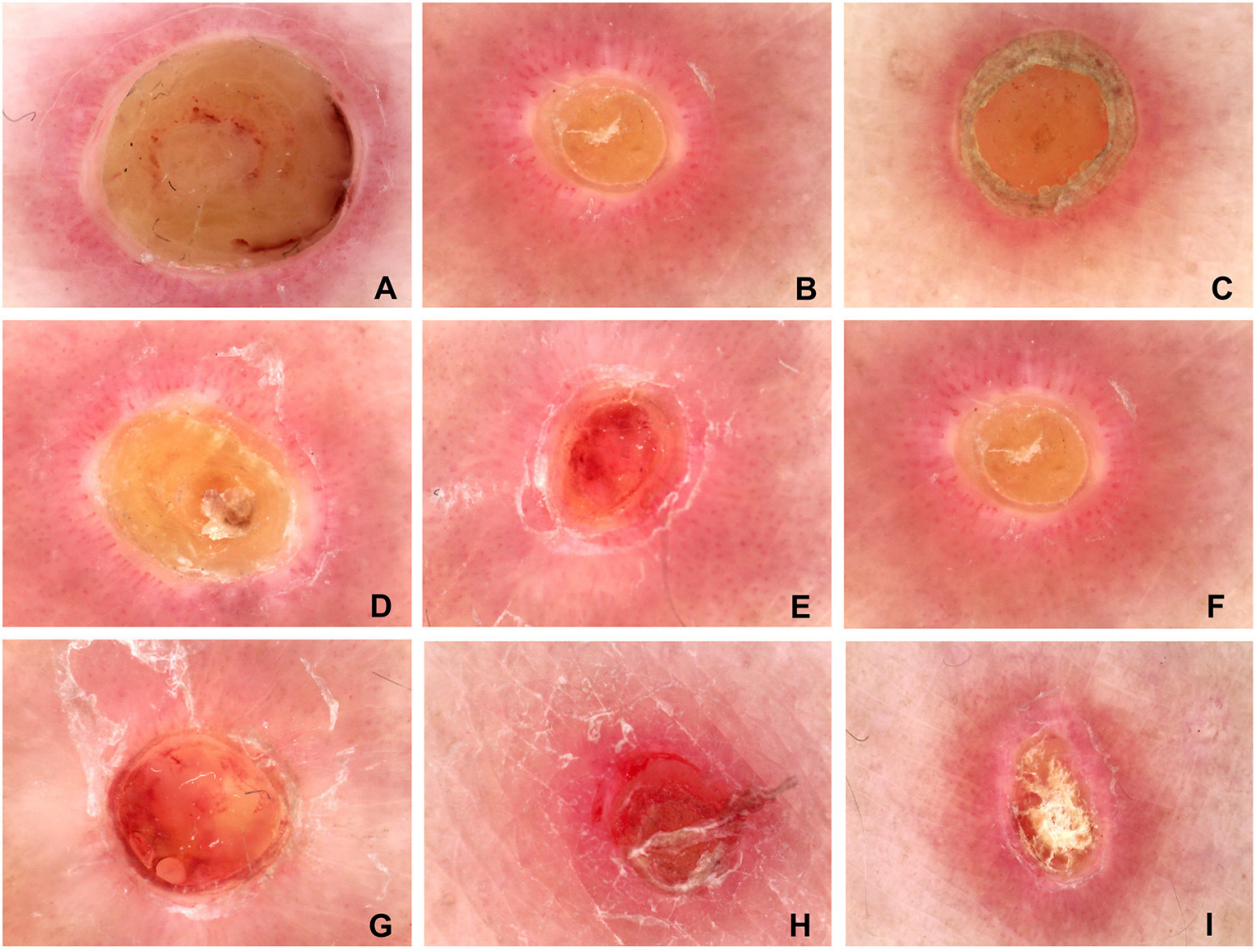
The common dermatoscopic features of the typical lesions from the 39 patients. **(A)** the yellow-brown homogeneous structureless area in the center of the lesion; **(B)** dotted and linear vessels distribution radially; **(C)** a dam shape uplift at the periphery of the lesion; **(D)** the white irregular halo surrounding the lesion; **(E)** a thin scale ring surrounding the lesion; **(F)** pink background; **(G)** orange background; **(H)** red background; **(I)** pigment structure surrounding the lesion. **(A–D)** the four common dermoscopic features of APD.

Dermatoscopic images reveal that the first four clear and consistent features corresponded to the histopathology changes of APD. The yellow-brown homogeneous structureless area in the center of the lesion was found to correspond to the collagen bundle that penetrates the epidermis vertically. The dotted and linear vessel distribution radially in the lesion was found to correspond to the perivascular inflammatory cells. The dam shape uplift at the periphery was found to correspond to the thickening of stratum spinosum and the form of cup-shaped depression of the epidermis by extruded collagen. This white irregular ring surrounding the lesion indicates pigment loss. The first three features also corresponded to a report of Emma Ormerod et al. (6). As described above, the dermoscopic features may be an aid in diagnosing APD and improve the rate of clinical diagnosis.

At present, there is no special treatment method for APD. Common treatment methods are to treat the primary disease to eliminate inducement. In this report, all patients were treated with oral antihistamines, topical glucocorticoid, and retinoic acid ointment combined with NB-UVB irradiation, and the efficacy was satisfactory. In conclusion, we found there are four common dermoscopic features of APD including a yellow-brown homogeneous structureless area in the center of the lesion, dotted and linear vessel distribution radially, and a dam shape uplift at the periphery, as well as a white irregular ring surrounding the lesion. There are three features, including the yellow-brown homogeneous structureless area in the center of the lesion, the dotted and linear vessel distribution radially, and a white irregular ring surrounding the lesion, which corresponds with the findings of Emma Ormerod et al. (6). These features are also similar to those previously described in three separate reports relating to seven cases of APD. In our report, we found a new dermoscopic feature: the dam shape uplift at the periphery. These findings could improve the rate of clinical diagnosis of APD.

According to our understanding, the kind of penetrating dermatosis, such as penetrating folliculitis, Kyrle disease, and elastosis perforans, have common features under the pathology, including parakeratosis keratotic plug or elastic fiber through the epidermis. We also summarized the differences in dermoscopic features between prurigo and APD. The dermoscopic features of prurigo show pale brown solitary raised papules with sticky white scabs, irregular white unstructured areas, and spotty, linear vascular foci. Different from the dermoscopic features of APD, there is no yellow-brown homogeneous structureless area in the center, no dam shape uplift at the periphery of the lesion, a white irregular halo surrounding the lesion, and the vascular arrangement is irregular. Therefore, hypothesize that the four dermoscopic features of APD described above are common dermoscopic features of penetrating collagen disease. The question of whether these four features can be defined simply as APD, requires further confirmation and study.

## Data Availability Statement

The original contributions generated for this study are included in the article/Supplementary Material, further inquiries can be directed to the corresponding author/s.

## Ethics Statement

Human research was approved by the Ethics Committee of Chengdu Second People's Hospital. The authors certify that they have obtained all appropriate patient consent forms and patients provided consent, confirming that their clinical information could be reported in journal articles.

## Author Contributions

All authors listed have made a substantial, direct and intellectual contribution to the work, and approved it for publication.

## Conflict of Interest

The authors declare that the research was conducted in the absence of any commercial or financial relationships that could be construed as a potential conflict of interest.

## References

[1] Millard PR Young E Harrison DE Wojnarowska F. Reactive perforating collagenosis: light, ultrastructural and immunohisto-logical studies. Histopathology. (1986) 10:1047–56. 10.1111/j.1365-2559.1986.tb02541.x 2430879

[2] Kim SW Kim MS Lee JH Son SJ Park KY Li K. A clinicopathologic study of thirty cases of acquired perforating dermatosis in Korea. Ann Dermatol. (2014) 26:162–71. 10.5021/ad.2014.26.2.162 24882969 PMC4037667

[3] Cerio R Calnan CD Wilson-Jones E. A clinic-pathological study of reactive perforating collagenosis: report of 10 cases. Br J Dermatol. (1987) 117:16–7. 10.1111/j.1365-2133.1987.tb11997.x

[4] Argenziano G Soyer HP Chimenti S Talamini R Corona R Sera F. Dermoscopy of pigmented skin lesions: results of a consensus meeting via the internet. J Am Acad Dermatol. (2003) 48:679–93. 10.1067/mjd.2003.281 12734496

[5] Goncharova Y Attia EA Souid K Protzenko O Koktishev I. Dermoscopic features of clinically inflammatory dermatoses and their correlation with histopathologic reaction patterns. Arch Dermatol Res. (2015) 307:23–30. 10.1007/s00403-014-1513-3 25297393

[6] Ormerod E Atwan A Intzedy L Stone N. Dermoscopy features of acquired reactive perforating collagenosis: a case series. Dermatol Pract Concept. (2018) 8:303–5. 10.5826/dpc.0804a11 30479861 PMC6246055

[7] Kittisak P Tanaka M. Dermoscopic findings in a case of reactive perforating collagenosis. Dermatol Pract Concept. (2015) 5:75–7. 10.5826/dpc.0502a13 26114057 PMC4462904

[8] Ramirez-Fort MK Khan F Rosendahl CO Mercer SE Shin-Chang H Levitt JO. Acquired perforating dermatosis: a clinical and der-matoscopic correlation. Dermatol Online J. (2013) 19:18958. 24010504

